# Dynamic changes in the mouse skeletal muscle proteome during denervation-induced atrophy

**DOI:** 10.1242/dmm.028910

**Published:** 2017-07-01

**Authors:** Franziska Lang, Sriram Aravamudhan, Hendrik Nolte, Clara Türk, Soraya Hölper, Stefan Müller, Stefan Günther, Bert Blaauw, Thomas Braun, Marcus Krüger

**Affiliations:** 1 Institute for Genetics, Cologne Excellence Cluster on Cellular Stress Responses in Aging-Associated Diseases (CECAD), 50931 Cologne, Germany; 2 Max Planck Institute for Heart and Lung Research, 61231 Bad Nauheim, Germany; 3 Institute of Biochemistry II, Goethe University Medical School, 60590 Frankfurt, Germany; 4 Center for Molecular Medicine (CMMC), University of Cologne, 50931 Cologne, Germany; 5 Venetian Institute of Molecular Medicine (VIMM), Department of Biomedical Sciences Padova, University of Padova, 35137 Padova, Italy

**Keywords:** Muscle atrophy, Denervation, Pulsed SILAC, Ubiquitination, Random forest

## Abstract

Loss of neuronal stimulation enhances protein breakdown and reduces protein synthesis, causing rapid loss of muscle mass. To elucidate the pathophysiological adaptations that occur in atrophying muscles, we used stable isotope labelling and mass spectrometry to quantify protein expression changes accurately during denervation-induced atrophy after sciatic nerve section in the mouse gastrocnemius muscle. Additionally, mice were fed a stable isotope labelling of amino acids in cell culture (SILAC) diet containing ^13^C_6_-lysine for 4, 7 or 11 days to calculate relative levels of protein synthesis in denervated and control muscles. Ubiquitin remnant peptides (K-ε-GG) were profiled by immunoaffinity enrichment to identify potential substrates of the ubiquitin-proteasomal pathway. Of the 4279 skeletal muscle proteins quantified, 850 were differentially expressed significantly within 2 weeks after denervation compared with control muscles. Moreover, pulse labelling identified Lys6 incorporation in 4786 proteins, of which 43 had differential Lys6 incorporation between control and denervated muscle. Enrichment of diglycine remnants identified 2100 endogenous ubiquitination sites and revealed a metabolic and myofibrillar protein diglycine signature, including myosin heavy chains, myomesins and titin, during denervation. Comparative analysis of these proteomic data sets with known atrogenes using a random forest approach identified 92 proteins subject to atrogene-like regulation that have not previously been associated directly with denervation-induced atrophy. Comparison of protein synthesis and proteomic data indicated that upregulation of specific proteins in response to denervation is mainly achieved by protein stabilization. This study provides the first integrated analysis of protein expression, synthesis and ubiquitin signatures during muscular atrophy in a living animal.

## Abstract (precis)

**Summary:** Comprehensive proteomic profiling of protein expression, synthesis and ubiquitination during skeletal muscle atrophy reveals that complex regulatory networks are activated during muscle wasting.

## INTRODUCTION

Numerous experiments have demonstrated that the metabolic and contractile properties of skeletal muscles can change rapidly in response to environmental influences (Cohen et al., 2015; Flück and Hoppeler, 2003). For example, physical exercise can induce muscle growth and activates signalling pathways that modulate mitochondrial activity, calcium homeostasis and muscle contractility (Matsakas and Patel, 2009). Conversely, ageing-associated diseases, such as cancer, type 2 diabetes mellitus and neurodegeneration, can lead to marked loss of muscle mass.

Skeletal muscle function is closely associated with motorneuron innervation. Reduced muscle recruitment, such as occurs during extended bed rest or spinal cord injury, results in a severe loss of muscle mass, which is also termed muscle atrophy (Gutmann, 1962; Jackman and Kandarian, 2004). Protein synthesis decreases, and processes that regulate protein degradation are enhanced during muscle wasting. Depletion of proteins is coordinated by both the ubiquitin-proteasome system (UPS) and autophagy-related processes (Lecker et al., 1999). Additionally, the members of the calcium-dependent nonlysosomal protease family (calpains) facilitate rapid degradation of myofibrillar proteins (Huang and Forsberg, 1998), and cellular organelles, such as mitochondria, are degraded via mitophagy, a specific form of autophagy (O'Leary et al., 2012; Vainshtein et al., 2015).

Unilateral sciatic nerve section is a well-established animal model of reduced neuronal stimulation (Goldspink, 1976; Gutmann, 1962; Medina et al., 1991). Previous studies demonstrated that denervation-induced atrophy elevates the cytoplasmic calcium concentration, activates the UPS and leads to remodelling of myosin heavy chain (MyHC) composition within individual muscle fibres (Ciciliot et al., 2013; Mitch and Goldberg, 1996; Zeman et al., 1986).

Analysis of the ubiquitin conjugation cascade in atrophying muscles demonstrated enhanced expression of several E2 conjugation enzymes and E3 ubiquitin ligases, each of which targets specific protein substrates for destruction via the proteasome. For example, the E3 ubiquitin ligases MURF1, atrogin-1 (MAFbx) and TRIM32 proteins are the key E3 ubiquitin ligases that mediate protein degradation during muscular atrophy (Bodine et al., 2001; Cohen et al., 2012; Gomes et al., 2001). Notably, MURF1 and TRIM32 are responsible for degradation of myofibrillar proteins, whereas atrogin-1 leads to degradation of MYOD, myogenin and the eukaryotic initiation factor 3 (eIF3).

Systematic microarray analysis of different muscle-wasting conditions, including starvation, diabetes, uraemia and denervation, has identified a specific set of genes called atrogenes, which are either up- or downregulated during muscle atrophy. So far, ∼120 atrogenes have been found, but it seems likely that this list is not complete (Lecker et al., 2004; Raffaello et al., 2006; Sacheck et al., 2007). Although mRNA expression studies have provided detailed insight into gene activity during atrophy, there have been few unbiased and systematic investigations of the changes in protein expression, protein synthesis and post-translational modifications during neuronal denervation of muscle tissue (Argadine et al., 2009; Goldspink, 1976; Ryder et al., 2015).

Recently, mass spectrometric (MS)-based proteomics has become an indispensable tool to measure proteins and their post-translational modifications. As a result of the development of powerful, high-resolution MS instruments, it is now possible to quantify the expression of vast numbers of proteins in complex biological samples (Cox and Mann, 2011; Mann et al., 2013). More importantly, the combination of MS-based proteomics with several enrichment strategies for specific modifications has enabled quantitative analysis of thousands of reversible protein modifications, including phosphorylation, acetylation and ubiquitination (Olsen et al., 2010). Public databases contain data on >500,000 post-translational modifications, demonstrating that individual proteins are highly engineered via diverse sets of modifications (Olsen and Mann, 2013).

The development of metabolic labelling approaches has facilitated accurate protein quantification and has been used to analyse protein turnover in both cell culture models and living animals (Schwanhäusser et al., 2009). The stable isotope labelling of amino acids in cell culture (SILAC) approach is based on metabolic incorporation of stable amino acids, for instance lysine and arginine, into the proteome of cultured cells or living animals (Krüger et al., 2008; Ong et al., 2002). To enable relative quantification of proteins between two different conditions, completely SILAC-labelled samples (condition one) can be combined with a nonlabelled cell population (condition two) and then subjected to combined MS analysis. The intensity of the nonlabelled (light) and labelled (heavy) peptide peaks can be used to assess the relative abundance of individual proteins of interest in each condition.

In pulsed SILAC experiments, labelled amino acids are added to the cell culture media for a short period of time. Newly synthesized proteins incorporate the labelled amino acids, and the incorporation rate can be used to compare relative protein turnover between two different conditions. SILAC labelling of living animals can be achieved by administration of specific diets containing a SILAC amino acid, such as ^13^C_6_-lysine, named Lys6. Uptake of the labelled food leads to incorporation of the labelled amino acid into the proteome, which enables protein synthesis to be monitored over time in living animals. For instance, a pulsed SILAC approach demonstrated that several lysosomal degradation substrates had reduced Lys6 incorporation and increased protein levels in a heart-specific atrogin-1-deficient mouse model (Zaglia et al., 2014). Direct incorporation of labelled amino acids can be used to compare the relative rates of protein synthesis in living animals (Nolte et al., 2015). However, it should be noted that relative isotope abundance, which reflects the ratio of natural and labelled lysine isotopes in the body (Claydon et al., 2012), must be calculated to estimate absolute protein turnover rates (*t*_½_).

Combinatorial analysis of relative changes in protein expression and synthesis might help to elucidate the complex regulatory network of protein synthesis and degradation that occurs during muscular atrophy and decipher the plasticity of skeletal muscle tissue. Thus, we combined *in vivo* SILAC expression profiling with a pulsed SILAC labelling approach based on time-dependent incorporation of Lys6 to quantify relative protein expression and synthesis in control and denervated gastrocnemius (GAST) muscles in a mouse model of sciatic nerve section. In addition, we used an immunoaffinity approach to enrich peptides containing a diglycine remnant (K-ε-GG) after tryptic digestion to identify potential targets of the UPS that undergo selective autophagy. The SILAC mouse spike-in approach enabled quantification of ∼4200 proteins, and administration of a Lys6-containing diet for 4, 7 or 11 days allowed calculation of ∼4700 incorporation rates for individual proteins in the murine GAST. Moreover, the identification of >2100 diglycine remnants provides the first quantitative signature of ubiquitination during muscular atrophy.

## RESULTS

### Denervation of the sciatic nerve rapidly alters protein expression profiles in the mouse GAST

**Fig. 1.**
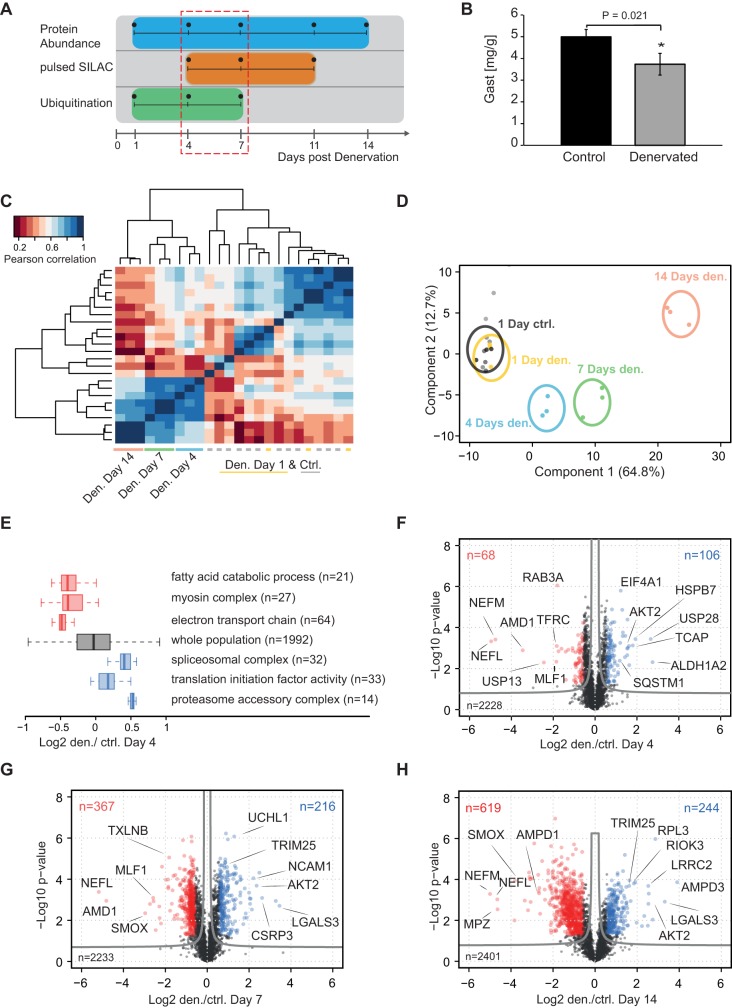
**Denervation-induced muscle atrophy in the mouse gastrocnemius muscle.** (A) Overview of the sciatic nerve section model. (B) Weight of the mouse gastrocnemius muscle (GAST, *n*=3) at 7 days after denervation. (C) Pearson correlation matrix of protein expression levels for all tested time points after denervation (biological triplicates). The heatmap based on Euclidean distance for the correlation matrix shows grouping of the control and denervated muscles. The correlation is based on log_2_ heavy/light (H/L) ratios shown in Table S1. (D) Principal component analysis protein levels showing clear separation of control and denervated muscle samples. (E) One-dimensional enrichment of GO terms at 4 days after denervation. Boxplots represent the log_2_ direct ratio distribution of proteins annotated with specific GO terms. (F-H) Volcano plots for day 4, 7 and 14, with correction for multiple testing by a randomization-based FDR calculation using a cut-off of 0.05 (fudge factor *S*_0_=0.1).

Unilateral section of the sciatic nerve was performed in wild-type C56BL/6J mice to induce muscle atrophy. The GAST was analysed at several time points over the first 14 days after denervation (Fig. 1A). The workflow of the MS analysis is indicated in Fig. S1A. At 7 days after sciatic nerve section, a significant loss (∼20% reduction; *P*=0.02) in muscle weight was observed compared with control muscles (Fig. 1B), similar to earlier reports (Sacheck et al., 2007). To investigate changes in protein expression during atrophy, we isolated the GAST after 1, 4, 7 and 14 days and performed relative protein quantification between control and denervated muscles. To enable accurate protein quantification, GAST muscles were isolated from Lys6-labelled SILAC mice. These animals were completely labelled with Lys6 over at least two generations and functioned as a spike-in standard to quantify nonlabelled peptides from control and denervated GAST muscles (Hölper et al., 2014). Each protein sample was mixed in a 1:1 ratio (based on total protein concentrations) with the SILAC GAST and subjected to in-gel digestion and LC-MS (liquid chromatography-mass spectrometry) analysis (Fig. S1A). Here, we identified 45,644 unique peptides representing 4279 identified protein groups at a false-discovery rate (FDR) <1% at the peptide and protein level (Table S1). Clustering analysis using the Pearson coefficient (*r*) and determining the Euclidean distances for different time points confirmed high reproducibility of our protein quantification between biological replicates (*n*=3; Fig. 1C). Likewise, principal component analysis (PCA) clearly confirmed the separation of protein intensities for control and denervated samples at different time points (Fig. 1D).

To identify whether groups of proteins that participate in the same pathways are regulated in a similar manner during atrophy, we performed one-dimensional enrichment of the log_2_ ratios of control and denervated muscles on day 4 after section. Boxplots of enriched gene ontology (GO) terms revealed that groups associated with ‘fatty acid catabolic process’ (GO: 0006631) and ‘myosin complex’ (GO: 0016459) were significantly downregulated at this early time point after denervation (Fig. 1E). Conversely, we observed that 32 proteins associated with the GO term ‘spliceosomal complex’ (GO: 0005681) and 14 proteins related to the ‘proteasome accessory complex’ (GO: 0022624) were upregulated at 4 days after denervation. Next, volcano plots were generated using two-sided *t*-tests to follow time-dependent protein changes in response to denervation. Although no significant changes in protein expression were observed on day 1 after denervation (Table S1), 174 and 583 proteins were significantly regulated in a different manner at 4 and 7 days after denervation, respectively, at a permutation-based FDR estimate of 0.05 (Fig. 1F,G). Over the longer term (14 days), denervation resulted in significantly differential expression of 863 proteins (FDR<0.05), indicating marked remodelling of the denervated GAST (Fig. 1H).

**Fig. 2.**
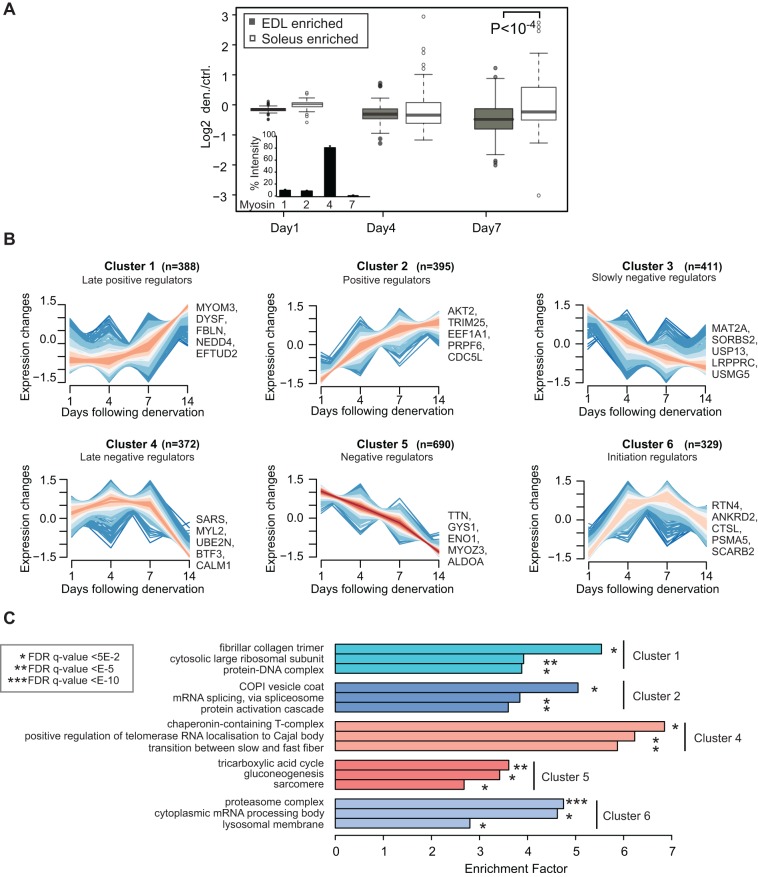
**Systematic characterization of protein expression changes during muscle atrophy.** (A) Overlap of protein expression data with soleus ‘slow’ and EDL ‘fast’ marker proteins. The boxplot analysis shows the comparison of EDL and soleus marker proteins between days 1 and 7. Inset: label-free quantification of myosin-1 (MYHC IIx), myosin-2 (MYHC IIa), myosin-4 (MYHC IIb) and myosin-7 (MYHC I). (B) *Z*-score normalized ratios were subjected to soft clustering with the mfuzz software tool. Six clusters represent different expression profiles upon denervation. (C) GO-term enrichment for clusters 1-5.

The GAST contains a mixture of type I and II fibres that express distinct sets of ‘slow’ and ‘fast’ myosin heavy chain (MyHC) proteins. A label-free protein quantification method based on unique MyHC peptides (Fig. 2A, inset) reflected the MyHC distributions reported in previous studies (Mänttäri and Järvilehto, 2005; Schiaffino and Reggiani, 2011). Next, we used a published list of proteins expressed specifically in the soleus and extensor digitorum longus (EDL) muscles to identify slow and fast fibre-specific proteins (Drexler et al., 2012), as shown in Table S2. Comparison of this catalogue with our protein expression data set revealed that 27% of fast fibre proteins were significantly downregulated after denervation of the GAST, whereas most proteins expressed in the slow soleus muscle were significantly upregulated on day 7 (Fig. 2A). This indicates that the slow type I fibres within the GAST are more resistant to denervation than type IIb fibres (Table S2).

### Protein expression profiling of denervated muscles reflects early remodelling activities

Sciatic nerve section is known to activate proteolytic pathways. Accordingly, we observed that 367 of the 2233 proteins quantified were significantly downregulated at 7 days after denervation (Fig. 1G). For example, the protein levels of neurofilament chains (NEFM, NEFL) and myelin proteins, such as myelin protein zero (MPZ) and the myelin basic protein (MBP), were significantly downregulated, indicating loss of neuronal innervation (Fig. 1F,H). By contrast, neuronal cellular adhesion molecule (NCAM) was ∼5-fold upregulated (*P*<10^−4^) at 7 days after denervation. Enhanced NCAM expression is associated with neuromuscular diseases and might be required to recruit axons to neuromuscular junctions (Cashman et al., 1987).

The histone deacetylase HDAC4 positively regulates genes associated with synaptogenesis and suppresses glycolytic enzymes after denervation (Tang et al., 2009). Here, we detected increased HDAC4 protein expression in denervated muscles, with no corresponding peptides detected in control muscles (Table S1).

The polyamine pathway synthesizes the metabolites spermidine and putrescine and is an important muscle homeostatic pathway. Reductions in the concentrations of enzymes involved in this pathway are closely associated with the induction of muscle wasting (Bongers et al., 2015; Lee and MacLean, 2011). Accordingly, we observed significant downregulation of spermine oxidase (SMOX) and *S*-adenosylmethionine decarboxylase proenzyme 1 (AMD1) in the denervated GAST, confirming the important roles of these enzymes in maintenance of skeletal muscle mass (Fig. 1H). Although polyamines can regulate Ca^2+^ flux, and cardiac hypertrophy is associated with increased polyamine concentrations, the exact physiological functions of those metabolites are poorly characterized and could represent an important area for future research on muscular atrophy (Lin et al., 2014).

Continuous muscle contraction is an energy-demanding process, and skeletal muscle possesses several metabolic pathways that produce energy efficiently. Consistent with earlier reports (Argadine et al., 2009; Lecker et al., 2004; Raffaello et al., 2006; Sacheck et al., 2007), we observed reduced abundance for nearly all metabolic pathways after denervation. However, some metabolic enzymes were upregulated after denervation, including 6-phosphogluconate dehydrogenase (PGD), an essential enzyme of the pentose phosphate pathway that converts 6-P-gluconate into ribulose 5-P and produces NADPH, which protects cells from oxidative stress (Budak et al., 2014). Given that denervation-induced muscle atrophy enhances the production of mitochondrial reactive oxygen species (ROS), increased PGD expression might be a direct response to increased ROS concentrations (Muller et al., 2007). Likewise, we found that the enzyme catalase (CAT), which also protects cells from oxidative damage, was significantly upregulated at the protein level on day 4 after denervation [fold-change denervated/control (den/ctrl): 2.4, *P*=1.4×10^−5^].

### Systematic clustering of protein categories reveals candidates with atrogene-like expression profiles after denervation

To enable more systematic analysis of differential protein expression, we conducted *z*-score normalization and supervised fuzzy clustering using the R-package mfuzz (Kumar and Futschik, 2007). Six clusters with different time profiles were identified. For example, cluster 6 represents a fraction of almost 400 proteins that were upregulated early and returned to baseline expression by day 14 following denervation. Notably, this cluster contains several lysosomal and proteasomal proteins (Fig. 2B,C). The 690 proteins in cluster 5 were constantly downregulated after denervation. To investigate whether protein clusters with similar expression patterns also exhibit similar cellular functions, we examined enrichment of specific GO terms. This analysis revealed over-representation of the terms ‘mRNA splicing’ (GO: 0000398) and ‘protein activation cascade’ (GO: 0072376) in cluster 2, whereas cluster 5 mainly contains sarcomeric proteins and proteins involved in carbohydrate metabolism and mitochondrial energy production (Fig. 2C).

**Fig. 3.**
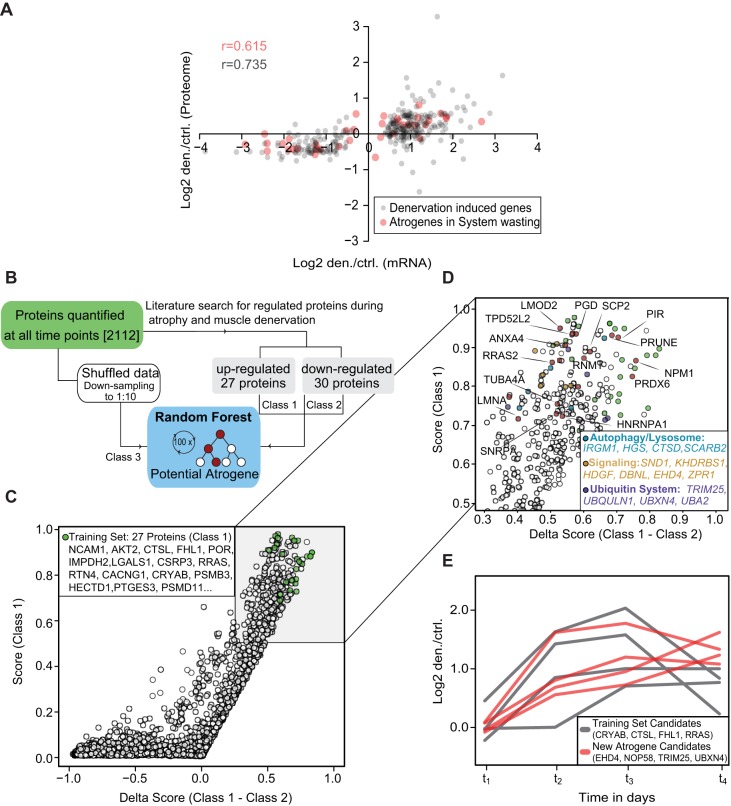
**Identification of proteins with atrogene-like signatures after denervation.** (A) Comparison of protein expression and previously published mRNA expression data. Grey circles represent the overlap of 720 denervation-induced mRNA changes with our proteomic data set. Red circles represent known atrogenes in systematic wasting and denervation. Pearson correlation ranged from 0.6 to 0.7. (B) Workflow of the random forest analysis. (C) Plotting of scores and delta scores based on random forest analysis, showing the distribution of all proteins. Green circles indicate selected upregulated protein candidates from the training set. (D) Higher magnification and selection of proteins with high probability scores. (E) Time profiles of selected training set proteins (class 1, grey lines) and proteins with similar enhanced expression profiles (red lines). Log_2_ fold-change den/ctrl was plotted against all four time points.

To relate transcript and protein levels directly, we compared our data set on differentially regulated proteins on day 4 with a previous cDNA microarray analysis describing mRNA expression changes in the denervated GAST muscle on day 4 (Sacheck et al., 2007). Of the 720 differentially regulated transcripts in the microarray analysis, we were able to overlap 418 proteins from our data set, resulting in *r*=0.74 (Fig. 3A, grey points; Table S3). The remaining 141 proteins that were identified to be regulated significantly on day 4 did not overlap with a differentially regulated mRNA in the microarray (Table S3).

Previous transcriptome studies identified a set of 49 genes that were consistently up- or downregulated in different muscle-wasting conditions, including denervation (Lecker et al., 2004; Sacheck et al., 2007). Comparison of these candidate mRNAs with our protein data set revealed congruent regulation of 40 proteins and mRNAs (*r*=0.62; Fig. 3A, red dots).

The random forest method classifies proteins based on decision trees, which are generated by specific training sets (Breiman, 2001). We selected 27 proteins from the literature that showed congruent increases in mRNA and protein expression after denervation for the training set (Fig. 3B). The candidates selected for the training set are listed in Table S4 and labelled with green circles in Fig. 3C. The proteins in the training set had an average probability score of 0.85, indicating congruent activation profiles during muscular atrophy. In total, we observed that 143 of the differentially expressed proteins in our data set had a probability score >0.7, indicating similar time-dependent protein changes to the proteins in the training set (Fig. 3D). To our knowledge, approximately half of these 143 proteins have not previously been described to be induced directly upon denervation; hence, they could be considered as new candidates involved in muscular atrophy (Table S4). Plotting the log_2_ protein ratios (den/ctrl) against time revealed similar kinetics for new candidates EHD4, NOP58, TRIM25 and UBXN4 (red lines) compared with selected proteins from our training set marked in grey (Fig. 3E).

Overall, this systematic analysis and clustering of quantified proteins demonstrated largely concordant protein regulation to that reported in earlier denervation studies. In addition, our analysis implicates other proteins that have not previously been recognized to be involved in early atrophy remodelling events. Whether these proteins are differentially regulated in other models of atrophy needs to be addressed in future studies.

### Protein expression of ubiquitin cascade and deubiquitinating enzymes

**Fig. 4.**
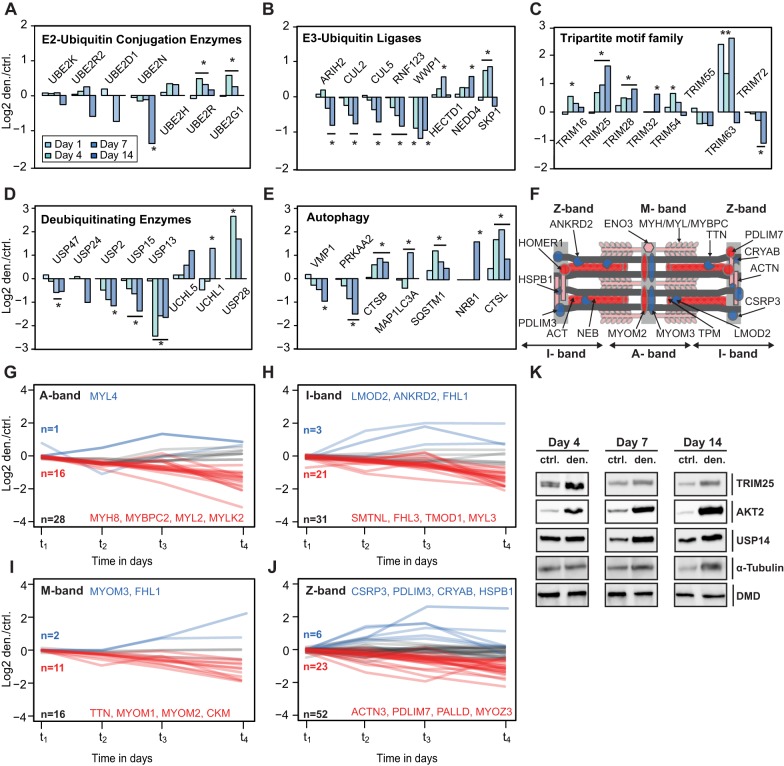
**Selected proteins associated with proteolysis and time-dependent protein changes in sarcomeric proteins.** (A-E) Bar diagrams showing protein ratios of selected E2-ubiqutin conjugation enzymes (A), E3-ubiquitin ligases (B), tripartite motif family (MURFs; C), deubiquitinating enzymes (D) and autophagy marker proteins (E) during the time course after denervation. (F) Schematic representation of the sarcomere; proteins labelled blue are upregulated and proteins labelled red are downregulated. (G-J) Protein expression time profiles of selected differentially expressed myofibrillar proteins. Log_2_ SILAC ratios were plotted against time [from day 1 to 14 after denervation (t_1_=day 1; t_2_=day 4; t_3_=day 7; t_4_=day 14)]. (K) Immunoblotting of lysates from control and denervated GAST using antibodies for TRIM25, AKT2, USP14 and tubulin. Dystrophin was used as a loading control. *Significant regulation (FDR cut-off=0.05). **Direct heavy/light SILAC ratio detected only in denervated muscle.

Based on GO terms, we identified 105 proteins associated with ubiquitin processing in the GAST; 18 of these proteins were upregulated after denervation. For instance, the ubiquitin-conjugating enzyme E2G1 (UBC7; also known as UBE2G2), an E2 conjugation enzyme related to endoplasmic reticulum-associated degradation (ERAD) (Friedlander et al., 2000), was upregulated on day 7 after denervation (fold-change den/ctrl 1.5, *P*=9.5×10^−5^; Fig. 4A). Notably, E2G1 has a modified ubiquitin conjugation domain that enables synthesis of K-48 chains, even in the absence of E3 ligases (Choi et al., 2015). Likewise, the E2 ubiquitin-conjugating enzyme R1 (UBE2R1; also known as CDC34) was slightly upregulated between day 4 and 7 after denervation (fold-change den/ctrl 1.5, *P*=3.7×10^−3^). CDC34 interacts with the SKP1, cullin-1 (CUL1) and F-box (SCF) complex and controls ∼20% of all ubiquitin-mediated protein degradation (Deshaies and Joazeiro, 2009). One function of CDC34 is catalysis of Lys48-linked polyubiquitination of the nuclear transcription factor inhibitor NFκBIA (Tan et al., 1999). However, it is most likely that the CDC34-SCF complex also ubiquitinates other targets during denervation that have not yet been elucidated. By contrast, two core components of cullin-ring-based E3 ubiquitin ligases (CUL2 and CUL5) were downregulated after denervation (Fig. 4B). The E3 ligases of the MURF family are required for rapid breakdown of sarcomeric proteins. Although MURF1 expression could not be detected in control muscles, the direct SILAC spike-in ratio revealed an ∼10-fold increase in MURF1/TRIM63 protein ratio over the time course of denervation, indicating strong upregulation of this E3 ligase during muscular atrophy (Fig. 4C; Fig. S1A). Interestingly, E3 ubiquitin ligase tripartite motif 25 (TRIM25), which has not previously been related to muscle atrophy, was also significantly upregulated, and we substantiated this regulation by performing western blot analysis (Fig. 4C,K; Fig. S2A).

Deubiquitinases are another important class of proteins that modulate proteolysis. Synthesis and disassembly of ubiquitin chains on target proteins is a vital process in all living cells. Dysregulation of deubiquitinases leads to severe human diseases, such as cancer and ataxia (Heideker and Wertz, 2015). Here, we identified 25 proteins with a direct or associated function in deubiquitination, which were regulated differentially in the GAST after denervation. For example, five members of the ubiquitin carboxyl-terminal hydrolase family, USP2, -13, -15, -24 and -47, were significantly downregulated 7 days after denervation (Fig. 4D). By contrast, USP28, a deubiquitinase involved in DNA damage responses (Knobel et al., 2014), was significantly upregulated (fold-change den/ctrl 6.3, *P*=3.6×10^−4^ on day 4). Likewise, USP14 was also slightly upregulated (fold-change den/ctrl 1.3, *P*=4.4×10^−3^), and we verified this trend by western blot analysis (Fig. 4K). USP14 is closely associated with the proteasome, and an earlier report described that USP14 inhibits the degradation of ubiquitinated proteins by the proteasome (Lee et al., 2010). Conversely, USP14 might also activate proteolysis by degrading ubiquitin chains on target proteins and thereby enhance gate opening of the 20S proteasome (Peth et al., 2009).

Finally, we identified that several autophagy marker proteins, including MAP1LC3A (Lc3-A; fold-change den/ctrl 2.1, *P*=7.1×10^−3^) and LAMP2 (fold-change den/ctrl 1.7, *P*=7.1×10^−5^) were significantly upregulated, indicative of proteolysis via the autophagy-lysosomal pathways after denervation (Fig. 4E).

### Parallel up- and downregulation of sarcomeric proteins during muscle atrophy

In addition to their essential role in muscle contraction, sarcomeres also contain proteins involved in cell signalling, control of gene expression, and protein turnover. To dissect sarcomeric substructures, we allocated the quantified proteins to their respective locations within the sarcomere. Previous studies characterized the distinct responses of thin and thick filaments during atrophy, with breakdown of thick filaments preferentially mediated by enhanced MURF1 activity (Cohen et al., 2009). The A-band consists of thick filaments. Most muscle-specific MyHCs, regulatory and essential myosin light chains (MyLCs) were downregulated after denervation (Fig. 4F,G). Conversely, we noticed that MYL4 and MYL12a were significantly upregulated after denervation (Table S1). Essential MyLCs interact with MyHC proteins to maintain the structural stability and modulate the motor function of myosins (Fig. 4G). MYL4 expression is restricted to the atria in normal conditions; MYL4 is expressed in skeletal muscle only during embryonic development and is downregulated in the neonatal period (Schiaffino et al., 2015). Endogenous re-expression of MYL4 in skeletal muscle is associated with regeneration, increased calcium sensitivity and enhanced cardiac contractility (Morano et al., 1996). It is possible that increased protein levels of both MYL4 and MYL12A in atrophying muscles reflect reduced loading during denervation, a condition that mirrors the low loads during embryonic development.

Next, we observed similar protein degradation trends for proteins associated with the I-band, a region containing thin filaments (Fig. 4H). However, some candidates, including ANKRD2, ANKRD23 and leiomodin 2 (LMOD2), were upregulated after denervation. The M-band stabilizes thick filaments and contains structural and metabolic enzymes. Consistently, most M-band proteins detected were downregulated after denervation, including myomesin-1 and 2 (Fig. 4I). Notably, myomesin-3 was the only detectable M-band protein that was significantly upregulated, indicating a different fibre type localization. The interaction of M-band proteins with obscurin, NBR1 and sequestosome-1 reflects their close association with protein quality control pathways (Gautel and Djinovic-Carugo, 2016). We observed early induction of the autophagy adaptor protein sequestosome-1 on day 4 after denervation (fold-change den/ctrl 2.3; *P*=4.2×10^−3^) and increased levels of NBR1 on day 14 (fold-change den/ctrl 2.2; *P*=4.3×10^−3^).

The Z-disc, an important structural unit that defines the lateral borders of the sarcomere (Knöll et al., 2011; Schiaffino and Reggiani, 2011), is a scaffold for titin and actin filaments and a hub for key signalling molecules, E3 ubiquitin ligases and mechanosensors. We identified 52 proteins associated with the Z-disc, and 55% of these proteins were differentially regulated significantly at 7 days after denervation.

The most abundant Z-band proteins are members of the α-actinin family. In agreement with earlier reports that showed α-actinin levels decreased in response to denervation and starvation (Cohen et al., 2012), we observed significant downregulation of α-actinin-2 and -3 from day 7 after denervation onwards. Conversely, HSPB7, CSRP3, α-crystallin (CRYAB) and PDLIM3 were more than 2-fold upregulated at 7 days after denervation (Fig. 4J). These changes indicate that rapid remodelling of Z-band structure occurs during atrophy, in support of the function of the Z-band as an important signalling intersection for sarcomere homeostasis.

### Assessment of protein synthesis during atrophy based on Lys6 incorporation

**Fig. 5.**
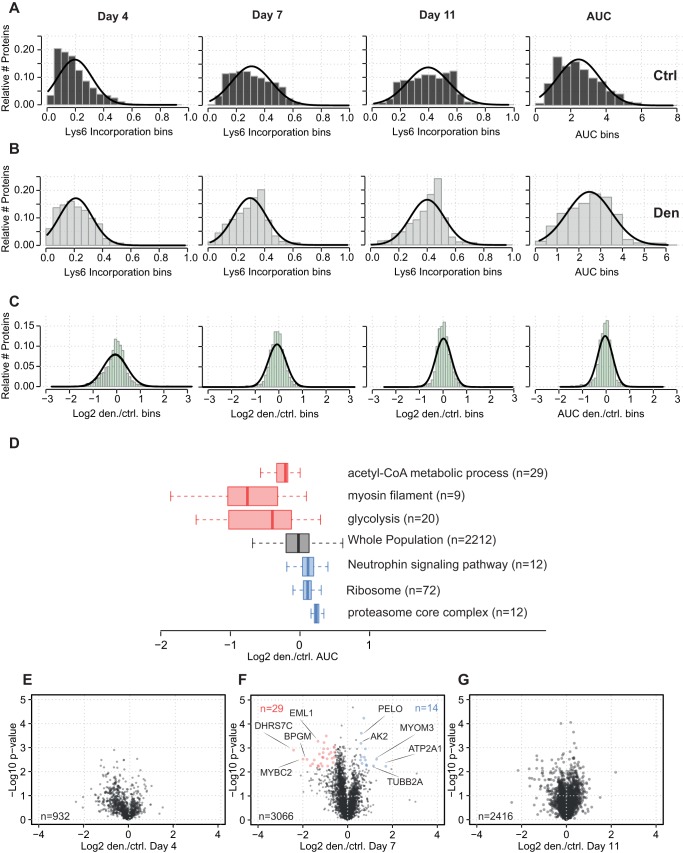
**Pulsed SILAC analysis of protein synthesis during atrophy.** (A) Relative Lys6 incorporation for control muscle over time. The percentage labelling was calculated as (H/L)/(H/L+1). The right panel in A shows the AUC for all time points. (B) Relative Lys6 incorporation for denervated muscle over time. (C) Ratio of Lys6 incorporation in control and denervated muscles. Log_2_ transformation ratios indicated a normal distribution. (D) One-dimensional enrichment and boxplot analysis show reduced Lys6 labelling of GO terms containing metabolic and myosin filament proteins (red boxes). Proteasomal proteins (blue boxes) exhibited increased Lys6 labelling. (E-G) Volcano plots of Lys6 incorporation in denervated and control muscles. One-sample *t*-test showing that significantly regulated proteins were observed only on day 7 (*q*<0.05). Day 4 samples were analysed as biological duplicates and day 7-11 as biological triplicates.

Changes in protein expression during muscle atrophy can be induced by alterations to the rate of protein degradation, the rate of protein synthesis, or both. To quantify protein synthesis during denervation-induced atrophy in control and denervated GAST muscles, we used a ^13^C_6_-lysine (Lys6)-containing mouse diet to label newly synthesized proteins (Fig. 1A; Fig. S1). Denervated mice were fed the Lys6 diet for 4, 7 or 11 days. Overall, the pulse-SILAC approach enabled Lys6 incorporation to be quantified for 4786 proteins (Fig. 5A,B; Table S1). Notably, earlier time points of SILAC labelling (days 1-3) were not feasible, as the intensities of the labelled peptides were barely detectable by MS. The areas under the curve (AUCs) for the comparison of Lys6 incorporation over time demonstrated similar labelling efficiency between the control and denervated muscles. Likewise, direct comparison of Lys6 incorporation also demonstrated an equal ratio distribution over time between control and denervated muscles (Fig. 5C). Furthermore, one-dimensional annotation enrichment of GO terms from the AUC values demonstrated enhanced Lys6 incorporation after denervation for proteins related to ‘neutrophin signaling pathway’ (KEGG AQ5: mmu04722), ‘ribosome’ [KEGG (Kyoto encyclopedia of genes and genomes): mmu03010] and ‘proteasome core complex’ (GO: 0005839) (Fig. 5D). Volcano plots revealed decreased or enhanced Lys6 incorporation for 43 proteins, but only on day 7 (*q*<0.05; Fig. 5E-G). For example, myomesin-3 and fast serca (ATPase sarcoplasmic/endoplasmic reticulum Ca^2+^ transporting 1; ATP2A1) had increased Lys6 incorporation, whereas the fast myosin-binding protein c2 (MYBPC2) had reduced Lys6 incorporation after denervation (Fig. 5F).

### Integration of Lys6 incorporation and protein expression during atrophy

**Fig. 6.**
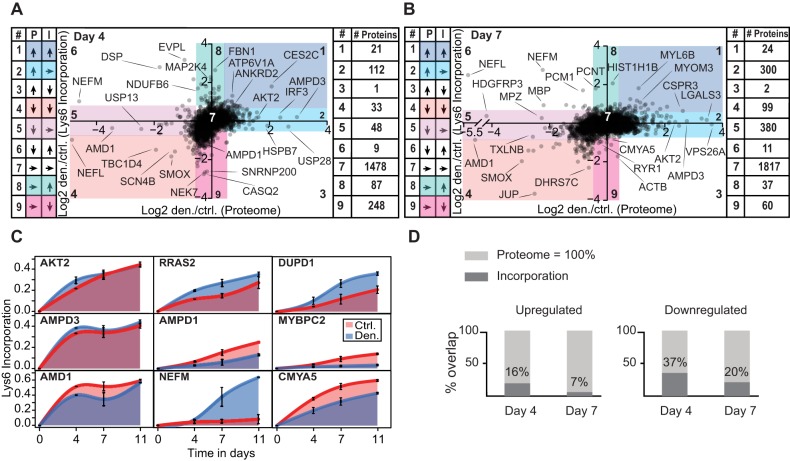
**Comparison of protein abundance and Lys6 incorporation.** (A,B) Scatter plots showing comparison of log_2_ ratios for protein expression levels and Lys6 incorporation in denervated and control muscles on day 4 (A) and day 7 (B) after denervation. The protein distributions within each sector (1-9) are listed on the left and right of each graph. (C) AUC plots of Lys6 incorporation in denervated and control muscles over time for selected proteins. (D) Bar diagrams illustrating the fractions of proteins that were up- or downregulated in the pulsed-SILAC and protein expression data sets.

Next, we determined whether denervation-induced changes in Lys6 incorporation are associated with altered protein expression (Fig. 6A,B,D). We demonstrated that only a minor fraction (∼22%) of downregulated proteins had reduced Lys6 incorporation (Fig. 6D). Most interestingly, 112 of the 134 proteins upregulated on day 4 and 300 of the 490 proteins upregulated on day 7 did not have altered Lys6 incorporation after denervation, suggesting that these proteins were stabilized, degraded, or both, at slower rates during atrophy than in control muscle (Fig. 6A,B, segment 2). For example, AKT2 kinase is a key signalling molecule that integrates several anabolic and catabolic pathways. We observed a ∼5-fold increase in AKT2 protein expression on day 7 (*P*=2×10^−4^), but Lys6 incorporation for this protein was not significantly different as indicated by the AUC curve for control and denervated muscles (Fig. 6C). Furthermore, 48 of the proteins that were downregulated on day 4 and 380 of the proteins that were downregulated on day 7 had unaltered or even increased Lys6 incorporation rates. For example, neurofilament proteins were severely downregulated on day 7, but had enhanced Lys6 incorporation rates. Such increases in protein synthesis might reflect a compensatory response to reduced protein expression levels.

### Regulation of diglycine remnants following denervation-induced atrophy

To provide additional insight into the mechanisms of denervation-induced atrophy and protein degradation, we enriched peptides with diglycine remnants in the GAST samples via an immunoaffinity approach on days 4 and 7 after denervation. As we used only lysine-labelled mouse tissues for SILAC quantification, we were restricted to use of the protease LysC, which specifically cleaves proteins after lysine residues, to ensure that all peptides could be used for quantification. Of note, tryptic digestion of lysine residues targeted by ubiquitin ligases was blocked; hence, trypsin digestion was used to obtain peptides with a diglycine remnant and at least one lysine residue per peptide. Following trypsin digestion, the SILAC peptide mixtures were separated by high pH reversed-phase liquid chromatography (RPLC) (Fig. S1A). In total, we generated 80 reverse phase fractions and pooled these fractions to create ten samples. Each sample was incubated with a cross-linked anti-diglycine antibody, subjected to an enrichment procedure and analysed by LC-MS (Udeshi et al., 2013), as illustrated in Fig. 1A and Fig. S1A.

**Fig. 7.**
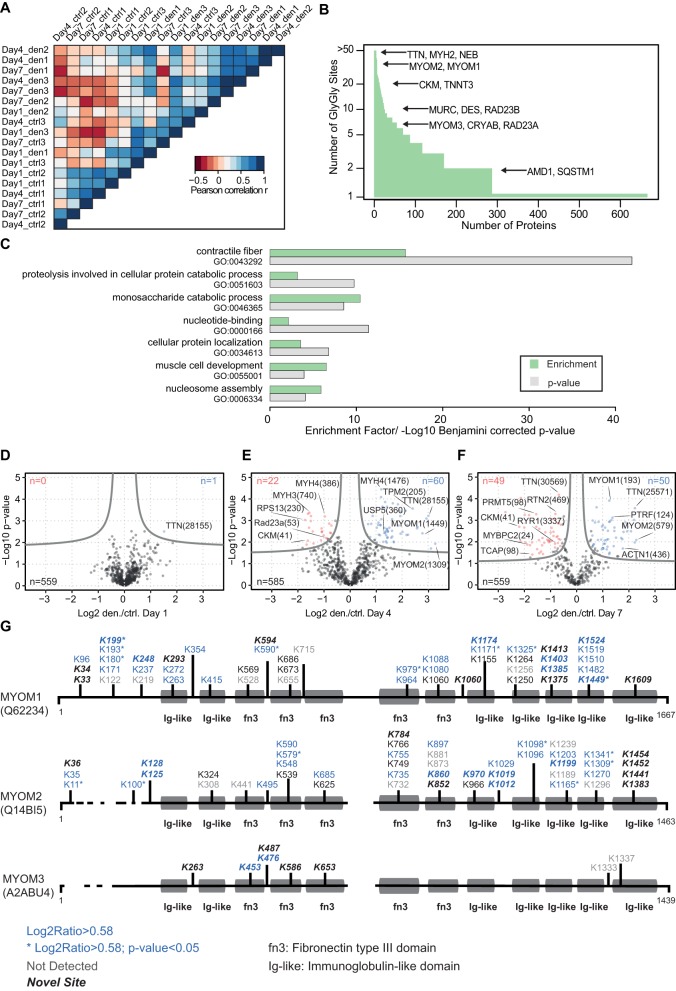
**Enrichment of diglycine remnants reveals increased myomesin-1/2 ubiquitination.** (A) Heatmap based on Euclidean distance for the correlation matrix shows grouping of the diglycine signatures of control and denervated muscles and demonstrates the high correlation between biological replicates. (B) Comparison of the number of diglycine sites per protein shows the distribution of the 2328 peptides with diglycine remnants identified by immunoaffinity enrichment and MS analysis. (C) Gene ontology term enrichment analysis of identified ubiquitination sites with DAVID (database for annotation, visualization and integrated discovery). The terms contractile fibres, nucleosome assembly and muscle cell development were significantly enriched (Table S1). The permutation-based FDR cut-off was set to 0.05. (D-F) Volcano plots of SILAC-quantified diglycine remnants in denervated and control muscles. The FDR was set to <0.05, with a fudge factor (*S*_0_) of 0.1. (G) Sequence alignment of three myomesin proteins and map of all diglycine peptides identified for myomesin-1, -2 and -3. Quantified diglycine sites are normalized according to their protein levels at each time point after denervation. Blue numbers indicate significant upregulation (FDR cut-off=0.05, fold-change >1.5).

Given that protein degradation via the UPS plays a fundamental role in muscle atrophy, it is important to assess ubiquitination patterns and changes during skeletal muscle atrophy (Sandri, 2013). The correlation heatmap separates control and denervated muscles by their ubiquitination pattern (Fig. 7A). Using a Lys-ε-Gly-Gly specific antiserum, we were able to enrich 2328 diglycine sites mapped to 667 different proteins (Fig. 7B; Fig. S2B). Moreover, 664 of these diglycine sites were newly identified, and GO analysis revealed that most of the diglycine sites identified originate from sarcomeric and metabolic proteins, reflecting the high abundance of these proteins in skeletal muscle (Fig. 7B,C; Table S5). In addition, a high number of diglycine sites were allocated to the UPS, ion transport or proteins involved in transcription and translation.

Volcano plot analysis indicated clear induction of regulated diglycine sites after denervation; 82 of the 585 (∼15%) diglycine modification sites were significantly up- or downregulated on day 4 after denervation (Fig. 7D,E; Table S1), and 99 of 559 (17%) on day 7 after denervation (Fig. 7F).

Next, we examined the ubiquitination pattern for the sarcomeric protein titin and mapped 418 diglycine remnants; 38 and 28 of these diglycine modification sites were significantly upregulated (*P*<0.05) on day 4 and 7 after denervation, respectively (Table S1; Fig. S2C). Notably, most sites upregulated at these early time points are close to the C-terminus, which is connected to the M-band. This ubiquitination gradient indicates that titin is proteolysed from the C-terminus towards the N-terminus. The initial breakdown of proteins localized within the M-band is further supported by the rapid downregulation of myomesin-1/2. Expression of both myomesin-1 and -2 was downregulated, and we detected that 16 of the associated ∼80 diglycine remnants were significantly enhanced after denervation (Fig. 7G). Conversely, we observed that myomesin-3 protein levels increased, but only two of the corresponding diglycine remnants (K453, K476) were enhanced after denervation.

## DISCUSSION

The trophic effects of neuronal innervation have been studied using a plethora of morphological, physiological and biochemical approaches (Gutmann, 1962). To extend our knowledge of alterations at the protein level, we determined the quantitative and temporal changes in protein expression, Lys6 incorporation and diglycine signatures during denervation-induced muscle atrophy in the mouse GAST. This analysis reveals the activation of several pathways and extends the catalogue of proteins that might contribute to the remodelling of the skeletal muscle proteome during denervation-induced muscle atrophy.

Previous transcriptional profiling studies revealed that a specific programme of genes is activated during muscular atrophy, with a set of ∼50 atrogenes commonly associated with catabolic states, including muscle denervation (Sacheck et al., 2007). Comparison of these genes with our data sets enabled us to generate a training set of 27 candidates by selecting known atrogenes that were congruently up- or downregulated in our data sets (Table S4). Systematic random forest analysis revealed similar ‘atrogene-like’ protein expression profiles for 143 upregulated proteins after denervation. Furthermore, upregulation of several proteins that participate in the UPS or autophagy, or function as cathepsin proteases after denervation was confirmed. In addition, we identified that a broad range of proteins, which have not previously been described to be involved in muscular atrophy, were differentially regulated after denervation. Hence, our protein expression data set provided a starting point from which we aimed to identify the individual proteins and networks that are potentially involved in early remodelling processes after denervation. However, it is necessary to investigate whether the known atrogenes and candidate proteins identified in this study are also differentially regulated in other muscle-wasting conditions.

Interestingly, we observed that several proteins demonstrated incongruent mRNA levels, protein levels and Lys6 incorporation after denervation. This suggests that increased mRNA expression, Lys6 incorporation, or both, can only explain the regulation of some proteins to an extent. For example, the neuronal cell adhesion molecule NCAM, protein kinase AKT2 and heat shock protein HSP7B were increased at the mRNA and protein levels, but their Lys6 incorporation did not change after denervation. Retinal dehydrogenase-2 (ALDH1A2) protein expression was significantly upregulated on days 4 and 7, with no increase in Lys6 incorporation. Moreover, altered *Aldh1a2* mRNA transcription was not detected in previous denervation studies (Sacheck et al., 2007). Conversely, nodal modulator 1 (*Nomo1*) mRNA expression was enhanced, with no change in NOMO1 protein expression or Lys6 incorporation. These data indicate that a variety of proteins are subjected to post-transcriptional and post-translational regulation during muscular atrophy. Although absolute turnover rates were not calculated, this study indicates that the increased expression levels of selected proteins observed in atrophying muscles are, at least in part, independent of altered mRNA levels or protein synthesis, suggesting that specific proteins are stabilized after denervation.

The breakdown of myofibrillar proteins is mediated by concerted activation of specific ubiquitin ligases, such as MURF1 and TRIM32. For example, proteins within the thick filaments are mainly targeted by MURF1, whereas the thin filament and Z-band proteins are ubiquitinated by TRIM32 (Cohen et al., 2009, 2012). The M-band structure is located in the centre of each sarcomere, and much attention has been focused on the dynamic regulation of M-band proteins during early muscular atrophy. Besides its essential structural function in organizing the lattice of thick filaments, the M-band also functions as a physiological sensor of stress-related conditions (Lange et al., 2005). This study confirmed that myomesin-1/2 are rapidly downregulated in atrophying muscle, accompanied by reduced Lys6 incorporation, and we observed 16 significantly enhanced diglycine modification sites on myomesin-1/2 after denervation (Fig. 7G). It seems reasonable to assume that the detection of diglycine modification sites on obscurin and FHL1 indicates that these M-band proteins are degraded by the E3 ubiquitin ligase MURF1 and TRIM32 during early atrophy. Recently, it has been shown that obscurin specifically interacts with myomesin-1, and it is tempting to speculate that this interaction influences the stability of myomesin-1 in fast fibres (Fukuzawa et al., 2008; Pernigo et al., 2017). Conversely, myomesin-3 protein expression and Lys6 incorporation were enhanced to a similar extent after denervation. In addition, diglycine remnant screening identified only two enhanced diglycine modification sites in myomesin-3. Previous studies demonstrated that myomesin-3 is localized within type IIa fibres, whereas myomesin-2 is mainly expressed in fast glycolytic type IIb fibres. Moreover, muscle fibres with distinct MyHC expression and metabolic activity exhibit differing responses to neuronal denervation (Schiaffino and Reggiani, 2011). Our data confirm that fast glycolytic fibres are more sensitive to denervation via selective degradation of proteins in type IIb fibres. In line with this suggestion, downregulation of myosin-4 was accompanied by enhanced diglycine modification sites compared with other MyHC proteins (Fig. S2D). Isolation of single muscle fibres and MS analysis might shed more light on the regulation of proteins during muscle atrophy and provide deeper insight into the early remodelling processes that affect individual fibre types and related structures (Murgia et al., 2015).

Rapid upregulation after denervation of several E3 ligases and autophagy-related proteins, including NBR1 and sequestosome-1, indicates that M-band proteins are most likely to be the first proteins targeted for breakdown during muscular atrophy. However, as the GAST contains different fibre types, more focused analysis of specific fibre types is required to determine the spatiotemporal gradient of protein degradation and the disassembly of myofibrillar structures in more detail (Volodin et al., 2017).

In addition, we observed an early upregulation of TRIM25 protein expression within 2 weeks of denervation, and our random forest analysis suggests TRIM25 as a new atrogene candidate protein. The functional relevance of TRIM25 during denervation remains largely unknown, although TRIM25 has been linked to breast cancer growth. TRIM25 activates the RIG1 receptor (retinoic-acid-inducible gene-I) via K63 ubiquitination, leading to enhanced NFκB activity (Gack et al., 2007; Horie et al., 2003). In this context, increased TRIM25 expression might reflect activation of inflammatory pathways. However, it seems reasonable to assume that TRIM25 also targets myofibrillar proteins during denervation. E3 ubiquitin ligases use different E2 conjugation enzymes to respond to signalling processes and modulate ubiquitin chain formation on target proteins. MURF1 has been reported to interact with several different E2 enzymes, including UBE2K, UBE2D and UBE2N, to form either K48 or K63 ubiquitin chains (Cohen et al., 2009; Kim et al., 2007). A recent enzyme-linked immunosorbent assay-based screening approach revealed that MURF1 interacts with UBE2D2/3 and UBE2E3 *in vitro* (Marblestone et al., 2013). However, the protein levels of these E2 enzymes were not upregulated after denervation; in fact, UBE2N was significantly downregulated. By contrast, UBE2R (CDC34) and UBE2G1 protein levels were upregulated after denervation, and these E2 enzymes could potentially modulate the function of E3 ligases during muscular atrophy. However, the functional relevance and mechanisms of ubiquitin chain formation remain unclear and should be addressed in future experiments. In addition, the quantitative diglycine modification site data sets generated in this study could represent a valuable resource to decipher the specific targets of E2/E3 ligases during muscular atrophy.

The giant protein titin is one of the most abundant proteins in skeletal muscle and connects the Z-band with the M-band in the sarcomere. Our analysis revealed a time-dependent increase in ubiquitination sites in titin, which spread from the C-terminus at the M-band towards the N-terminal region at the Z-band (Fig. S2C). It is tempting to speculate that ubiquitination of titin starts at the M-band, where several ubiquitin ligases modulate the turnover of M-band-associated proteins (Gautel, 2008). It will be interesting to determine whether ubiquitin ligases such as MURF1 and other tripartite motif-containing proteins are responsible for this gradient of titin ubiquitination; such studies will help us to understand the sequential order of protein degradation during muscular atrophy. A recent study revealed biphasic breakdown of myofibrillar proteins in denervated tibialis muscles in mice. The initial step is catalysed by TRIM32 and induces enhanced ubiquitination of desmin filaments. Although not significantly altered, we detected nine diglycine remnants on desmin during the 2 weeks after denervation. These diglycine remnants might reflect the onset of desmin breakdown and subsequent myofibrillar degradation after denervation (Volodin et al., 2017).

Accordingly, our proteomics analysis also demonstrated enhanced protein levels of several cathepsin proteases and autophagy markers, such as MAP1LC3A, sequestosome-1 and NBR1. Formation of lysosomes is required for degradation of extra- and intracellular material. Lysosomal-associated membrane protein (LAMP2) is one of the most abundant lysosomal proteins (Gonzalez et al., 2014). LAMP2 and the lysosomal membrane protein LIMP II (also known as SCARB2) were upregulated after denervation. Targeting LIMP II to lysosomes is mainly organized by the clathrin-associated adaptor protein complex, and we also demonstrated that the adaptor protein AP3 was significantly upregulated after denervation. Moreover, overexpression of LIMP II expanded early and late endosomes and lysosomes in cell culture (Kuronita et al., 2002). Therefore, our data indicate that LIMP II-dependent enlargement of lysosomes might also occur during early muscular atrophy.

The random forest analysis revealed that pirin (PIR) protein expression levels increased after denervation. This non-haem iron-binding protein is a coregulator of NFκB (Wendler et al., 1997; Liu et al., 2013). Following translocation of NFκB proteins into the nucleus, pirin associates with coregulators and binds to NFκB target genes (Pahl, 1999). For example, pirin interacts with NFκB50-BCL3 dimers to enhance the DNA-binding activity of this complex (Dechend et al., 1999). Pirin levels increase in response to oxidative stress, and oxidation simultaneously activates the iron atom of pirin. Thus, pirin functions as a redox sensor to facilitate DNA binding of transcription factors under oxidative stress. Indeed, denervation of skeletal muscle increases oxidative stress, which is an important signal for transcriptional activation of target genes (Abruzzo et al., 2010). Pirin expression is also enhanced in superoxide dismutase (*Sod*)-deficient mice. Although the functional relevance of pirin during denervation-induced atrophy is largely unknown, pirin might act as a redox sensor that activates pro-inflammatory pathways during muscular atrophy.

In conclusion, this integrated proteomic analysis demonstrates that marked regulation of protein expression, protein synthesis and protein ubiquitination occur in response to denervation-induced atrophy in skeletal muscle. By combining existing microarray data sets with our proteomics approach, we are confident of identifying new networks and relevant signals implicated in the pathobiology of muscular atrophy.

## MATERIALS AND METHODS

### Generation of SILAC mice

C57BL/6 mice (male, 9 weeks of age) were fed a ^13^C_6_-lysine (Lys6)-containing mouse diet (Silantes GmbH, München, Germany) to generate the labelled SILAC mouse colony, as previously described (Krüger et al., 2008). Administration of a diet containing Lys6 leads to a complete exchange of naturally occurring ^12^C_6_-lysine (Lys0) over one generation (Hölper et al., 2014). Isolated tissues and extracted proteins from SILAC mice were used as a heavy ‘spike-in’ standard for accurate quantification of the experimental conditions.

### Muscle denervation and tissue lysis

Section of the left sciatic nerve was used to induce muscular atrophy (Lecker et al., 2004). After isolation of the control and denervated gastrocnemius (GAST) muscles, samples were snap frozen in liquid nitrogen. Frozen muscle tissue was ground to a fine powder using a mortar and pestle. Tissue powder from all experimental conditions was extracted with SDS lysis buffer (4% SDS in 100 mM Tris/HCl, pH 7.6). Lysates were homogenized, heated at 70°C for 10 min and clarified by centrifugation. Protein concentrations were determined using the Bio-Rad DC assay.

### LysC protein digestion

For analysis of the proteome, 20 µg of nonlabelled and labelled protein extracts were mixed and loaded onto a 4-12% Bis-Tris gel (Invitrogen, Carlsbad, CA, USA), separated by SDS-PAGE and stained using the Colloidal Blue staining kit (Invitrogen). Each lane of the gel was cut into ten slices, and proteins were digested in-gel with LysC (Wako Pure Chemical Industries, Richmond, VA, USA) overnight at 37°C, as previously described (Shevchenko et al., 2006). Briefly, the gel pieces were destained in 50 mM ammonium bicarbonate and ethanol, reduced with 10 mM dithiothreitol (DTT) at 56°C for 45 min, carbamidomethylated with 55 mM iodoacetamide in the dark for 30 min at room temperature, and digested with 12 ng/µl LysC at 37°C overnight. The next day, digestion was stopped by acidification with trifluoracetic acid (TFA), and peptides were extracted from the gel pieces with increasing concentrations of acetonitrile (ACN). The organic solvent was vacuum evaporated using a SpeedVac concentrator plus (Eppendorf, Hamburg, Germany), and peptides were desalted using C18-based Stop and Go Extraction Tips (Rappsilber et al., 2007).

### Trypsin digestion and peptide purification

For analysis of diglycine-containing peptides, 7.5 mg of the SILAC spike-in was mixed with equal protein amounts of nonlabelled control and denervated samples. After precipitation with acetone for 2 h at −20°C, samples were resuspended in urea buffer (6 M urea, 2 M thiourea in 10 mM HEPES, pH 7.6). Proteins were reduced with 5 mM DTT for 30 min at room temperature, carbamidomethylated with 10 mM iodoacetamide for 30 min in the dark at room temperature and subsequently digested with LysC at an enzyme:substrate ratio of 1:100 for 3 h at room temperature. Protein samples were diluted to 2 M urea using 50 mM ammonium bicarbonate and digested overnight at room temperature using sequencing-grade trypsin (Promega, Fitchburg, WI, USA) at an enzyme:substrate ratio of 1:100. After digestion, peptides were acidified with formic acid (FA) and subsequently desalted using a 500 mg C18 Sep-Pak SPE cartridge (Waters, Milford, MA, USA). C18 cartridges were preconditioned with 5 ml of ACN, followed by 5 ml of 50% ACN, 0.1% FA and 15 ml of 0.1% TFA. After acidification and clarification by centrifugation, samples were loaded onto the conditioned C18 cartridges and washed with 20 ml of 0.1% TFA. Bound peptides were eluted thrice with 2 ml of 50% ACN/0.1% FA and concentrated on a SpeedVac concentrator plus (Eppendorf) to ∼100 µl.

### Peptide fractionation by high pH RPLC

Off-line high pH RPLC was performed using an XBridge BEH300 C18 3.5 µm column on an ÄKTA Purifier (GE Healthcare Life Sciences, Little Chalfont, UK). Fractionation was performed as described previously (Udeshi et al., 2012). Specifically, concentrated samples were resuspended in RPLC buffer A (5 mM ammonium formate in 2% ACN, pH 10) and injected at a flow rate of 0.25 ml/min. Peptides were fractionated using a 64 min gradient that started by increasing the concentration of RPLC buffer B (5 mM ammonium formate in 90% ACN, pH 10) to 8% at a rate of 1.1% B/min, followed by a 38 min linear gradient from 8 to 27% B. The gradient was then ramped successively to 31% B at 1% B/min, 39% B at 0.5% B/min and 60% B at 3% B/min. During the entire fractionation, the flow rate was 0.25 ml/min. In all, 75-80 fractions were collected, and pooled in a non-contiguous manner to obtain ten fractions that were concentrated to ∼100 µl on a SpeedVac concentrator.

### Enrichment of ubiquitinated (K-ε-GG) peptides

The anti-K**-**ε-GG antibody was obtained from the PTMScan^®^ ubiquitin remnant motif kit (Cell Signaling Technology, kit #5562). The antibody was cross-linked before use, as described previously (Udeshi et al., 2012). Antibody beads were washed twice with 1 ml of 100 mM sodium borate (pH 9.0) at 4°C and then cross-linked using 1 ml of 20 mM dimethyl pimelimidate (DMP) on a rotating wheel for 30 min at room temperature. Cross-linked beads were washed twice with 1 ml of 200 mM ethanolamine (pH 8.0) at 4°C, then cross-linking was blocked with 1 ml of 200 mM ethanolamine on a rotating wheel for 2 h at 4°C. Subsequently, the antibody beads were washed twice with 1 ml IAP Buffer (50 mM MOPS, pH 7.2, 10 mM sodium phosphate, 50 mM NaCl) at 4°C and resuspended in IAP buffer. Concentrated peptide fractions obtained from RPLC were resuspended in 1 ml IAP buffer and incubated with cross-linked antibody beads (divided equally for ten fractions) for 2 h on a rotating wheel at 4°C. After immunoprecipitation, beads were washed twice with 1 ml ice-cold PBS and 1 ml ice-cold water. Bound K**-**ε-GG peptides were eluted twice with 100 µl of 0.15% TFA and purified using C18-based Stop and Go Extraction Tips.

### Lys6 incorporation

Mice were denervated as described above and immediately switched to a mouse diet containing ^13^C_6_-lysine (3 g/day) until they were sacrificed at specific time points for extraction of the GAST muscles from both control and denervated legs. Tissue lysis and in-gel protein digestion with LysC were performed as described above.

### LC-MS/MS analysis

RPLC of peptides and MS/MS analysis were performed using an Easy nLC 1000 UHPLC coupled to a QExactive mass spectrometer (Thermo Fisher Scientific, Waltham, MA, USA). Peptides were resuspended in Solvent A (0.1% FA), picked up with an autosampler and loaded onto in-house made 50 cm fused silica emitters (75 µm diameter) packed with 1.9 µm C18-AQ Reprosil Pur beads (Dr Maisch GmbH, Ammerbuch, Germany). Samples were loaded at a flow rate of 750 nl/min. A 150 min segmented gradient of 10-38% Solvent B (80% ACN in 0.1% FA) over 120 min and 38-60% Solvent B over 7 min at a flow rate of 250 nl/min was used to elute peptides. Eluted peptides were sprayed into the heated transfer capillary of the mass spectrometer using a nano-electrospray ion source (Thermo Fisher Scientific). The mass spectrometer was operated in a data-dependent mode, where the Orbitrap acquired full MS scans (300-1750 *m*/*z*) at a resolution (R) of 70,000 with an automated gain control (AGC) target of 3×10^6^ ions collected within 20 ms. The dynamic exclusion time was set to 20 s. From the full MS scan, the ten most intense peaks (*z*≥2) were fragmented in the high-energy collision-induced dissociation (HCD) cell. The HCD normalized collision energy was set to 25%. MS/MS scans with an ion target of 5×10^5^ ions were acquired with a resolution R=35,000, with a maximal fill time of 120 ms, isolation width of 1.8 *m*/*z*, capillary temperature of 280°C, and spray voltage of 1.8 kV.

### MS data processing

MS raw data were analysed using MaxQuant software version 1.4.1.2 (Cox and Mann, 2008). Peptides were searched using the Andromeda search engine (Cox et al., 2011) using the mouse UniProt database containing 73,921 entries. Multiplicity was set to 2, and Lys6 was set as the labelled amino acid to quantify SILAC peptide pairs. LysC was chosen as the digestion enzyme for protein identification and trypsin for ubiquitinated peptide identification, both with allowance of cleavage N-terminal to proline. The maximal number of missed cleavages allowed was two. Cysteine carbamidomethylation was set as a fixed modification; and methionine oxidation, N-terminal protein acetylation and Gly-Gly addition to lysines as variable modifications. The maximal mass tolerance was 20 ppm, initial precursor ion mass deviation, 7 ppm and MS/MS tolerance, 0.5 Da. An FDR of 1% and minimal peptide length of seven amino acids were applied for peptide identifications. For quantification of SILAC peptides, a minimal ratio count of two was applied. Ubiquitinated site identification and quantitative information were obtained from the MaxQuant GlyGly(K) sites table. Statistical data analysis and *t-*tests were performed using Perseus (version 1.3.8.3).

### Data analysis

Soft clustering of *z*-score normalized log_2_ fold-change of proteomic data was performed using the R-package Mfuzz (Kumar and Futschik, 2007). The parameter *c* (number of clusters) was set to six, and the parameter *m* (fuzzifier) was estimated (based on the data) to be 2.54. The AUC of the incorporation-time profile was calculated using the R-package MESS. To analyse proteome and ubiquitination data, a two-sided *t*-test was performed using a permutation-based FDR of 0.05 (fudge factor *S*_0_=0.1) in Perseus. Statistical analysis of incorporation data was performed using one-sample tests in Perseus. Multiple testing correction was performed using the R-package qvalue, calculating *q*-values at an FDR <0.05.

### Random forest analysis

A random forest approach using the comprehensive caret (classification and regression training) R package (Kuhn et al., 2008) was used to identify potential atrogenes in the proteomics data set. First, we performed a literature search and screened transcriptomics data for known atrogenes, and classified the corresponding proteins into the following two classes: Class 1, upregulated upon denervation; and Class 2, downregulated following denervation. Then, we generated a negative data set (Class 3) by shuffling the complete proteomics data set (time course experiment after denervation). We tuned the trained random forest by maximizing Cohen's kappa (statistic) separately for Class 1 and Class 2 against Class 3. Tuning of the random forest was performed on the parameter mtry (e.g. randomly selected features at each split). Given that the negative data set could introduce bias by chance, we trained a total of 50 random forest for each class, and the probabilities for Class 1 and 2 were averaged, respectively. For visualization, we plotted the probability for Class 1 versus the Delta Score (Class 1−Class 2).

### Western blotting and antibodies

Equal amounts of protein from each muscle lysate (∼60 µg) were separated using the TGX stain-free sodium dodecyl sulphate-polyacrylamide gel electrophoresis (SDS-PAGE) system from Bio-Rad (Hercules, CA, USA). Proteins were transferred onto a polyvinylidene difluoride membrane using the Bio-Rad TransBlot Turbo system and detected with the following commercial antibodies: rabbit polyclonal anti-Akt (Cell Signaling, #9272, 1:1000), rabbit monoclonal USP14 (Cell Signaling, #11931, 1:1000), mouse monoclonal anti-α-tubulin (Sigma Aldrich, T9026, 1:500) and rabbit monoclonal anti-TRIM25 (Abcam, ab167154, 1:000). An anti-dystrophin antibody (Dys1, Novocastra, NCL-DYS1) was used as a loading control. Proteins were visualized using a chemiluminescence system (Amersham ECL Prime) from GE Healthcare.
